# Self-Care Experiences of Empty-Nest Elderly Living With Type 2 Diabetes Mellitus: A Qualitative Study From China

**DOI:** 10.3389/fendo.2021.745145

**Published:** 2021-11-18

**Authors:** Xiaoyan Lv, Doris S. F. Yu, Yingjuan Cao, Jinghua Xia

**Affiliations:** ^1^ Department of Nursing, Qilu Hospital, Cheeloo College of Medicine, Shandong University, Jinan, China; ^2^ Nursing Theory & Practice Innovation Research Center, Shandong University, Jinan, China; ^3^ School of Nursing, LKS Faculty of Medicine, The University of Hong Kong, Hong Kong SAR, China; ^4^ Department of Nursing, Beijing Jishuitan Hospital, Beijing, China

**Keywords:** self-care, empty-nest elderly, Type 2DM, qualitative study, China

## Abstract

**Background:**

The number of type 2 diabetes mellitus (T2DM) cases among empty-nest elderly increases with increasing aging in China. Self-care plays an important role in preventing and reducing adverse outcomes of diabetes; however, few studies focus on self-care experiences of empty-nest elderly with T2DM.

**Objective:**

To explore self-care experiences for a chronic disease among empty-nest elderly patients with T2DM in mainland China.

**Methods:**

A descriptive phenomenological design was used in this study. Semi-structured interviews were conducted for 15 empty-nesters with T2DM. Interviews were implemented in department of endocrinology at a tertiary teaching hospital located in Shandong province, east of China.

**Results:**

The participants were poorly adept with monitoring their blood glucose and lacked the ability to deal with abnormal blood glucose levels. Most participants had a good relationship with medication and physical activity. Living without children was perceived as a benefit that improved dietary management and is a disadvantage in terms of economic and emotional support and access to medical resources. Elderly empty-nesters also lacked knowledge about diabetes and paid little attention to potential complications.

**Conclusion:**

Empty-nest elderly patients with T2DM value medication compliance and lifestyle modification more than blood glucose monitoring, complication prevention, and coping with negative emotions. Friends and spouses play indispensable roles in patients’ self-care motivation and maintenance. Diabetes education on self-care, access to medical resources, and social support is needed for better diabetes management.

## Introduction

Aging is a worldwide phenomenon, more than 1.5 billion people will be aged 65 and over, accounting for 16% of the total population in 2050 (1). The concurrent increase in the nuclear family, as well as the aging population, implies that a number of older adults will have an empty-nest experience. Empty-nest elderly refers to the elderly with no children or those who do not live with children, but with a spouse, or alone (2). The problem with empty-nesters has become more acute in China. In 2010, the number of empty-nesters aged 60 years and above accounted for 50% of the total number of the elderly, which is expected to reach 90% by 2030 (3). The one-child policy, acceleration of urbanization, and imbalance of economic development are considered as major catalysts contributing to this situation (4).

The diabetes prevalence rate is also rapidly increasing among the elderly population of China. China has the largest number of diabetics worldwide aged 65 and older, along with a deepening social aging, more than 95% of these individuals were type 2 diabetes mellitus (T2DM) (5). An empty-nest status has a significantly adverse influence on different areas of elders’ health such as cognitive ability, psychological health, and physical health; and empty-nest elders had significantly poorer health than non-empty-nest elders (6) owing to less economic support, spiritual consolation, and daily care from adult children.

Self-care is considered to have an important role in improving the quality of life and delaying the occurrence of complications (7). A high level of self-care is associated with improved blood sugar control and reduced all-cause mortality (8, 9). Ma conducted a cross-sectional study to explore the impact of empty-nesting on self-care among older adults with diabetes (10). The results indicated that self-care, in terms of diet, physical exercise, blood glucose monitoring, medication, and foot care, were poorer among empty-nesters than those living with children. The results may be related to the disconnection between empty-nesters and adult children, relatives, and friends, which can lead to an inadequate knowledge of DM, self-care, and a limited number of information sources.

The experiences of self-care among empty-nest elderly with T2DM have not been investigated. This study calls for more attention to the self-care needs of empty-nesters with T2DM. A more comprehensive understanding of their experiences with managing diabetes can shed light on their unique needs and corresponding care advancement. Understanding what empty-nesters realize about their condition and what influences their motivation, is needed for health care professionals to recognize barriers to effectively managing and controlling diabetes.

## Materials and methods

### Study Design

A descriptive phenomenological design was used in this study. The main methodological consideration of descriptive phenomenology is to describe, explore, and analyze a phenomenon in adequate depth and breadth so as to elucidate the comprehensive picture of interest (11). Descriptive phenomenological design has become a popular method for understanding the life experiences of a unique group of individuals and its meanings and interactions with others and the environment (12).

### Participants and Recruitment

Purposive sampling was used to recruit empty-nest older adults with T2DM from the department of endocrinology in the Qilu Hospital of Shandong University, China. The inclusion criteria were as follows: (i) aged 60 years and above; (ii) having a medical diagnosis of T2DM; (iii) an empty-nest status as defined by not living with children for at least 12 months and a frequency of children’ visits less than once a week. Interviews were conducted until the point of saturation was reached.

### Data Collection

Semi-structured interviews ranged from 40 to 60 min and were conducted in a private room in the department of endocrinology. A self-developed, semi-structured interview guide, based on literature on the challenges of diabetes-related self-care and expert opinion (a head nurse, endocrinology), was developed. Key questions were asked as follows: How do you manage blood sugar in your daily life?; How much do you know about diabetes management and the prevention of complications?; and How does your family support you in your disease management? To protect the privacy of participants, the interviewees negotiated the time with participants and completed the interview in a private meeting room after the end of routine treatment and when their condition was stable. The interviews were implemented by the first author, who is a postgraduate student, with diabetes care experience and good communication skills, but has not yet participated in patient care. All interviews were conducted face-to-face and audio-recorded with the permission of participants. Memos or notes were also written and used for data analysis.

### Ethical Considerations

The research was approved by the Human Research Ethics Committee of School of Nursing, Shandong University (No.2016-R-23). Written consent was obtained from all participants. Verbal consent was obtained to use a digital voice recorder prior to the commencement of each interview. Personal information and interview content were kept anonymous and confidential. All participants had the right to withdraw from the interview at any time.

### Data Analysis

All interviews were transcribed by students in nursing school. JH checked the scripts against the audio recordings. XY and JH analyzed five transcripts independently, and themes were checked by the research team for consensus. Afterwards, the coding scheme was used only for the remaining transcripts. New codes were inserted when necessary.

### Rigor

In order to guarantee the reliability and rigor of this study, Guba and Lincoln standards were adopted, including credibility, transferability, conformability, and dependability (13). The researcher in this study had been practicing in the department of endocrinology and had established a good relationship with patients. During the interview, participants’ words were fed back to them for confirmation and to assure the credibility of the data. To improve data transferability, detailed descriptions about research steps were applied for a better understanding of the data. An audit trail was put in place to demonstrate the process of data interpretation and induction and to increase the conformability. For dependability, the interview manuscripts were reviewed by members of the research team, and themes were extracted and discussed within the team.

## Results

A total of 15 empty-nesters with T2DM were recruited to participate in the in-depth interview. The characteristics of the participants are shown in **Table 1**. The mean age of the participants was 65 years (standard deviation [SD] = 5.03). The mean duration of diabetes was 11 years (standard deviation [SD] = 6.54).

**Table 1:** Demographic data of participants (n=15).

Participant	Age (years)	Gender	Living pattern	Treatment	Duration of diabetes(years)	Number of comorbidity	Caregiver
1	69	Female	Live with spouse	Oral drugs	9	1	No
2	77	Female	Live with spouse	Oral drugs	11	2	No
3	60	Female	Live with spouse	Oral drugs	2	2	No
4	62	Female	Live with spouse	Insulin injection	14	3	No
5	72	Female	Live with spouse	Insulin injection and oral drugs	10	3	No
6	62	Female	Live with spouse	Oral drugs	6	2	No
7	64	Female	Living alone	Insulin injection	2	1	No
8	64	Female	Live with spouse	Insulin injection	10	3	No
9	60	Male	Live with spouse	Oral drugs	20	2	No
10	73	Female	Live with spouse	Insulin injection	20	2	Yes
11	61	Male	Living alone	Insulin injection and oral drugs	15	4	No
12	61	Female	Live with spouse	Oral drugs	20	4	Yes
13	65	Male	Live with spouse	None	1	1	No
14	63	Male	Live with spouse	Oral drugs	20	4	No
15	64	Female	Live with spouse	Insulin injection	10	5	No

**Figure 1:**
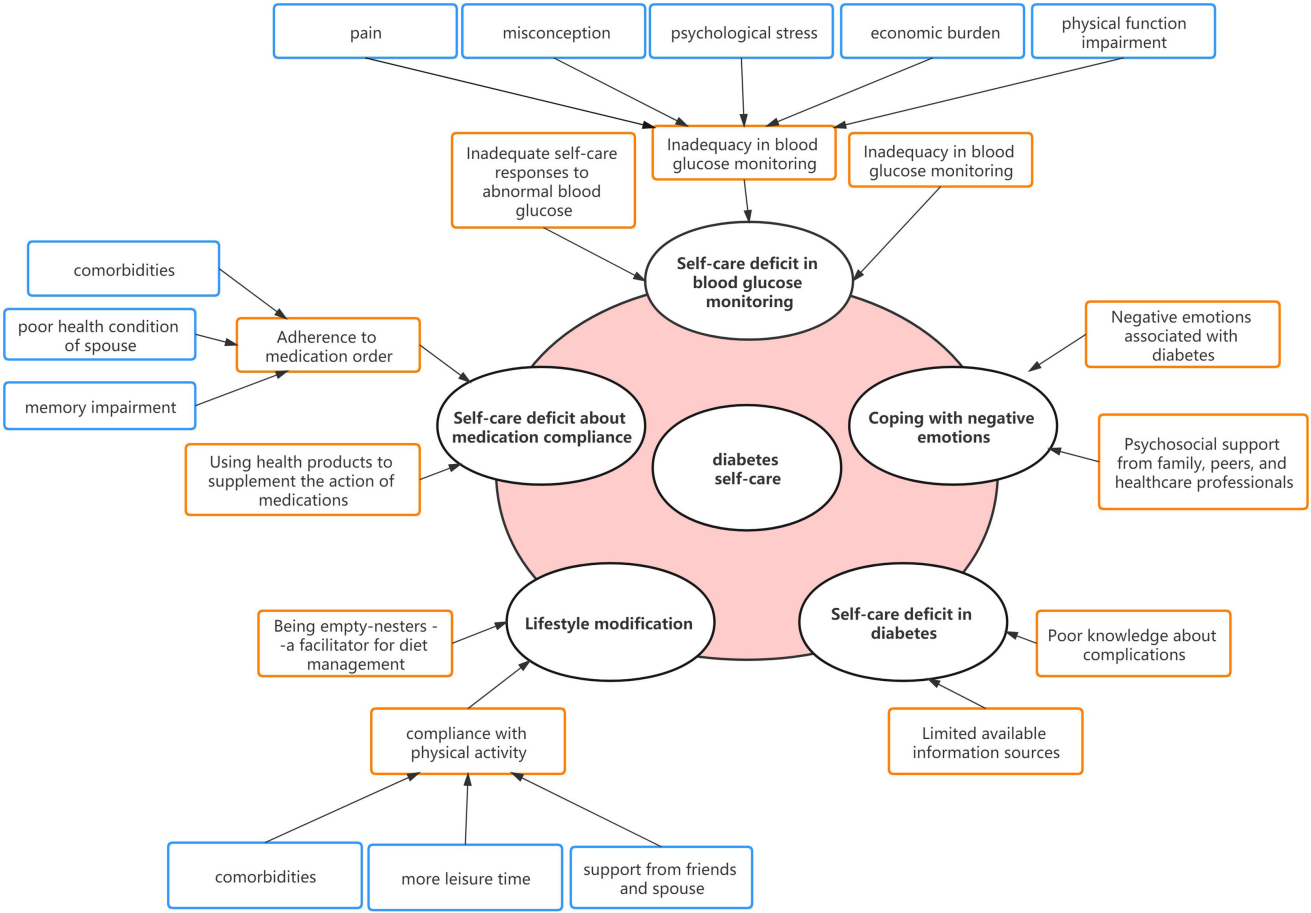
Thematic presentation of self-care experiences in empty-nesters with diabetes.

**Figure 2:**
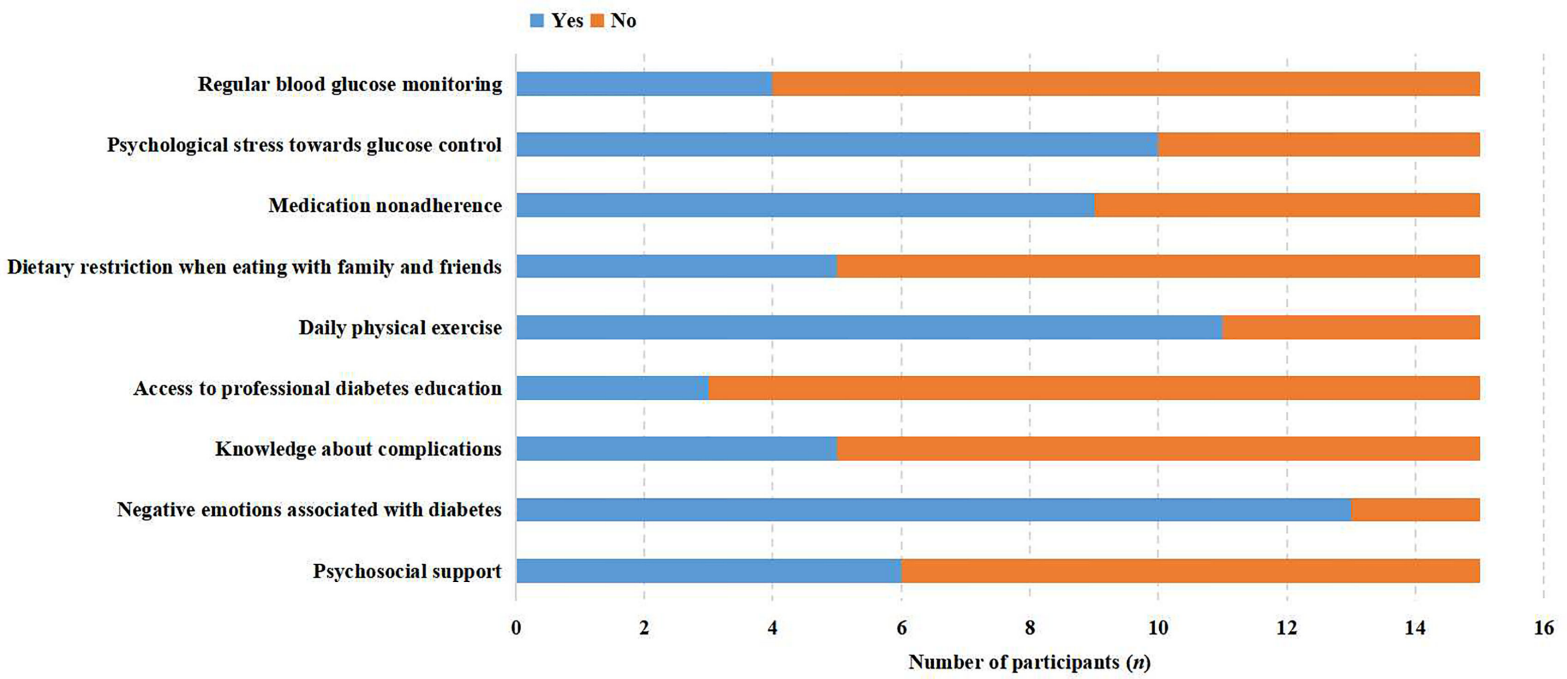
Participant responses to each question.

Five major themes were identified to illustrate the self-care experiences of empty-nesters with T2DM including a self-care deficit in blood glucose monitoring, with medication compliance, lifestyle modification, in preventing diabetic-related complications, and coping with negative emotions. Themes and subthemes are presented in **Figure 1** and the participant responses to each question were presented as positive or negative feedback in **Figure 2** through stacked bar charts. Responses of participants associated with themes and subthemes were described in **Supplementary File**.

### Theme 1: Self-Care Deficit in Blood Glucose Monitoring

#### Inadequacy in Blood Glucose Monitoring

The participants were found to have poor compliance with blood glucose monitoring. The results indicated that the mean interval between two blood glucose tests was 15 days to six months. Regular monitoring was limited to the time when participants were newly diagnosed with T2DM or the disease exacerbated, as they paid more attention to the management of their disease during such a critical period. Otherwise, they had irregular monitoring and took blood glucose measurements only when they felt uncomfortable symptoms, such as dizziness and heart palpitations.

*I will record blood sugar for a few days if health care providers ask me to do this. Recording is decreased after this period. (P4)*

*I measure blood glucose about once a month when it occurs to me. (P12)*

*I tested my blood sugar every day when I first suffered from diabetes. It’s different now. I will not examine my glucose until I feel uncomfortable. (P15)*

#### Barriers for Regular Blood Glucose Monitoring

The main barriers for regular blood glucose monitoring are as follows: (1) the pain associated with blood glucose monitoring; (2) a misconception that the monitoring was not needed when they had a desirable blood glucose control; (3) increased psychological stress associated with unsatisfactory results; (4) economic burden caused by blood glucose test strips; (5) physical function impairment such as inadequate eyesight to engage in the monitoring. The following are several quotes for illustration.

*Sometimes I do not want to test my blood sugar; I’m in a terrible temper when the reading rises. (P4)*

*The test strips were used up so fast, 50 pieces were gone in a few days. It is pretty expensive to buy strips, and needles can be used repeatedly for another time, after first disinfecting with alcohol. (P5)*

*I know my condition of blood sugar control, and I don’t think it’s necessary for me to measure it regularly. (P9)*

*I suffer complications with my eyes. It is difficult for me to install the blood taking needle. There is nobody available to help me do this. (P13)*

*It’s very painful for me to measure my blood sugar, so I never test myself. (P14)*

#### Inadequate Self-Care Responses to Abnormal Blood Glucose

When patients have physical discomfort caused by hyperglycemia, such as leg dancing, dry mouth or headache, they do not have the skills to deal with this situation, and usually do not seek the help of healthcare providers until they cannot bear it. They are used to these experiences and think it is not a problem to worry about. Going to the hospital is full of challenges for them, because the hospital has made a lot of electronic reform, which requires certain experience and health literacy. They can hardly adapt to the rapid development of the new mode of medical treatment and examination without help. The following are quotes for illustration.

*Perhaps because I am old, going to the hospital to see a doctor is really a big challenge for me, now the hospital and the city is developing so fast and use many intelligent equipment. I will not come to the hospital until my condition is particularly serious. (P1)*

*I do not know what else to do when the glucose reading exceeds 16mmol. That’s terrible. (P12)*

### Theme 2: Self-Care Deficit About Medication Compliance

#### Adherence to Medication Order

Most participants took medicine regularly according to a doctors’ prescription, especially those who received insulin treatments. A few patients stated that doctors did not tell them how to take the different drugs and lead them to take the drugs incorrectly. Participants are accustomed to asking friends or a spouse, rather than healthcare providers, about confusion related to taking medication. The advice of friends and spouses was more accessible than that of healthcare providers. It was a universal phenomenon for them to forget whether they took their pills or not, owing to too many kinds of drugs and memory impairment, even though they were reminded by a spouse. There was also poor compliance with the medication for patients whose spouses suffered from diseases which lead to difficulties in self-care (such as hemiplegia caused by stroke), because they were too busy taking care of their spouse to take their medication regularly.

*I often forget to take my medicine and insulin injection, although I have been unwell for a long time. Sometimes I fail to bring an insulin pen with me when I go out. (P4)*

*When I encounter confusing questions, I will ask my spouse and friends first, because it is more convenient to communicate with them, and the doctor is far away from me. (P5)*

*My spouse is hemiplegic. Sometimes when I am busy taking care of him, I forget to take my medicine. I am the only one in the family who can’t cope with it at some time, not to mention also managing diabetes. (P12)*

#### Using Health Products to Supplement the Action of Medications

Apart from formal medication prescribed by the doctors, some patients were relying on health products to control blood sugar. Empty-nesters had a tendency to spend lots of money purchasing these health products when they heard effective remarks. They have a great desire to cure diabetes and hope they can recover and reduce the burden on their families and children, rather than become useless to them. Peer recommendations make them full of expectations about the effects of health care products. The lack of adult children’s judgment and supervision of diabetes management behavior was also one of the reasons for patients to buy health products.

*As I get older, I feel useless. I can’t help with my children’s affairs, and I don’t want to bother my children with trifles. What I hope for the most is that there are some ways to cure diabetes, so that I can suffer less and be less trouble for my family. (P2)*

*She (a neighbor) told me that her blood glucose level dropped after taking health products without any diabetes medication intake. I spent 5 thousand RMB to buy it for its good effect. I took it for 2 months, and there was not any improvement in my blood sugar. I haven’t finished it yet. (P8)*

### Theme 3: Lifestyle Modification

#### Being Empty-Nesters-A Facilitator for Diet Management

Most patients expressed that they pay great attention to their daily diet, including eating a fixed amount on a fixed schedule, and one third stated that they did not follow any dietary restrictions. They can better follow dietary modifications when they are eating at home, by themselves, or with a spouse. When children visited and had meals with them, they would override any dietary restrictions to adhere to the food preferences of their children, they did not want to eat differently when co-dining with their children. In addition, some adult children lacked knowledge about diabetes and believed that the dietary restrictions were merely limited to sweet foods. They bought and persuaded their parents to eat food that did not include the proper dietary restrictions. Patients did not resist the temptation and would break dietary compliance. This indicated that living alone or with a spouse was beneficial to reduce dietary non-compliance.

*I eat the same as the children do when they are back home, and I eat more coarse grains for breakfast and dinner. (P4)*

*It is easy to manage my diet without living with children. Some of my children like eating meat; also my grandchildren need to grow up healthy and require foods that I am restricted from. (P9)*

*I don’t have much control overeating out with other people. If you adhere to dietary restrictions when eating out, it is considered that you are picky with food and out of tune with others. (P15)*

#### Compliance With Physical Activity

Most patients expressed they had more leisure time because they live without adult children. Eleven participants indicated that they would do 30 minutes to 2.5 hours of outdoor exercise almost every day. Their willingness to exercise, and the duration, were affected by disease (two patients had their meniscus removed, two had a history of myocardial infarctions, one suffered from arthritis, one had an ankle joint injury). Some patients rarely exercised outdoors because of multimorbidity. Participants believed that both their friends and their spouse played a positive role in terms of supervising and assisting outdoor activities.

*I don’t exercise regularly. I usually do housework at home. I go out for a walk when there is nothing to do at home. I used to do exercise and now, it is difficult for me to walk too much since I broke my ankle five years ago. (P3)*

*I walk 5 or 6 laps, at least, around the square every afternoon. I go out at 2 p.m. and am back home at about 5 p.m. I insist on walking 2.5 hours a day, approximately, unless there is severe weather. (P10)*

*I often go out for a walk with my friends, or with my spouse. They give me a lot of companionship and encouragement. I think it’s hard for me to keep going out for exercise almost every day without their company and supervision; they are important to helping me have the motivation to exercise. (P15)*

### Theme 4: Self-Care Deficit in Diabetes

#### Limited Available Information Sources

Empty-nester patients were limited in their perception of diabetes. Most patients learned about diabetes from local television programs (such as *The Road to Health and Honyaradoh*), or Internet, and rarely participated in health lectures organized by hospitals or communities. Many patients indicated that they did not trust the health education done in the community. They considered that the professional capability of community healthcare providers was limited, and that the teaching repeated what the book said, which was tedious and ambiguous and a mere task of the government. At the same time, they lacked the information source of hospital health education popularization activities. The majority of reliable knowledge came from healthcare providers in hospital when they were attending visits.

*I haven’t heard of any community lectures, and I wouldn’t attend them even if I had. I attended a lecture before, which was given by an old nurse. I couldn’t understand what she was talking about. She left after the lecture without any interaction. Some of them are just there to make money. The quality of lectures is poor, and I don’t want to listen again. (P1)*

*My mother has diabetes. I learned about diabetes from her; moreover, I receive knowledge from HCPs when I go to the hospital or community clinic. (P3)*

*I watch TV and listen to lectures at home. “The Road to Health” is the only China Central Television talk service program focusing on the public’s physical and mental health awareness and advocating a healthy life. I’d like to watch this at home. (P4)*

#### Poor Knowledge About Complications

They put more focus on the control of blood sugar, and little was known about the prevention and management of complications. Many patients indicated that they did not know the complications that came with diabetes, such as foot lesions and diabetic retinopathy before their hospital admission. Several patients realized that their eyes would be impacted by diabetes. However, they knew less about how to prevent eye complications and the methods by which they could take care of. A few patients examined the condition of eyes regularly, though many suffered from eye complications already. Most patients checked complications when they were required to do so by doctors in the hospital.

*I didn’t know the complications of diabetes before, nor did I know how to deal with them. I have no discomfort. (P7)*

*I used to know little about the complications of diabetes, but I’ve got eye complications now, and it’s hard to see things (frown). (P9)*

### Theme 5: Coping With Negative Emotions

#### Negative Emotions Associated With Diabetes

Emotional fluctuations and negative emotions are common in empty-nester elderly individuals with T2DM. They felt stressed, upset, and confused, particularly when their diabetes was under poor control with increasing symptoms and when their blood glucose was fluctuating to a great extent, and they had no idea how to deal with it. Patients considered that it was unbalanced for them when compared with non-diabetics. Most patients agreed that diabetes self-care costs them a lot of energy and time. There were also numerous restrictions that made it hard for them to relax.

*The last time I sick, I was thinking about how hard it is for humans from birth to death. I suffer living with diabetes. (P8)*

*I get upset and don’t want to communicate with others when my blood sugar is not under control (P10).*

*It is suffering to have diabetes, you must pay attention to your diet, physical activity, and insulin injection. It is imbalanced compared with the responsibilities other people have (sigh). (P15)*

#### Psychosocial Support From Family Peers, and Healthcare Professionals

Patients shared most of their negative emotions with their spouse and friends with diabetes, instead of with their children. Most patients indicated that friends and spouses gave them spiritual motivation, sympathy, and stress relief. Some participants expressed that they accepted little support from their spouse because of limited understanding about their disease and needs. Most patients chose to bury their negative emotions and considered that if they poured their frustration out to others, it would make them annoying. Two patients stated that they would go to the hospital for psychological counseling when they felt depressed. The majority of patients seldom discuss negative emotions with health care providers. In addition to psychological support, they need more life and medical support when their spouse’s physical condition is declining, their own disease control is poor, and they are far away from the hospital.

*He (spouse) spends most of his time playing cards, watching TV, and doing other things that he is interested in, rather than paying attention to my disease. (P2)*

*When I’m feeling down, I talk to my friends. They encourage me to do better. They also patiently listen to me. It makes me feel like I’m not alone in the face of diabetes. (P3)*

*I adjust myself sometimes or express my feelings to my daughter. She blames me all the time. She is busy and stressed with her life, and I don’t want to trouble her too much … Our children treat us well and always buy us something to eat. Those things are unsuitable for me. The most common thing they say to me is to take my medicine on time and to exercise more. They always say that. (P8)*

## Discussion

To our knowledge, this qualitative study is the first to explore the experiences of self-care among empty-nest elderly patients living with T2DM. Our findings show that empty-nest elderly patients lack the understanding and proper implementation of self-care behaviors. They had relatively strong compliance with dietary modifications, physical activity, and medication. Yet, self-care related to blood glucose monitoring, complication prevention, and coping with negative emotions, was compromised. Decreased body functioning and limited access to help from adult children are factors that affect the self-care of empty-nest diabetic elderly. Instead, friends and spouses play a positive role in helping these patients maintain self-care motivation and compliance.

Participants did better in diet and medication adherence, which may be owing to the obvious and direct effects on blood glucose control. Living without children seems to be an advantage to dietary control. Following a healthy diet is a well-recognized factor that impacts one’s ability to achieve glycemic targets and prevent or delay the onset of severe diabetes complications (14). Family conflicts about diet were identified as a stressor for patients when their dietary restrictions impacted household meals; therefore, they tended to accommodate others’ tastes when preparing a meal for the whole family (15, 16). However, it is available to choose the lifestyle they prefer for patients who are living without children at home. It provides a great opportunity for elders to enjoy their leisure time and spend their energy taking care of themselves (6). Zhang indicated that the health promoting lifestyle of empty-nesters was better than that of non-empty nesters (17). The possible reason was that empty nesters didn’t have the pressure of work or the burden of taking care of children, so they had more time and energy to communicate with others and learn how to keep healthy. However, non-empty-nesters were more likely to be caregivers for their children and offspring (17).

The participants in this study lacked self-management knowledge and available sources of health information, which was consistent with previous studies (18–21). Diabetes is a chronic condition whose long-term prognosis is highly dependent on the self-care behavior of the affected people. Diabetes self-management education has become an essential part of diabetes care. Studies have shown that education in primary health care settings improved self-management behavior, reduced the burden of disease and the risk of diabetes-related complications (22–26). In the past ten years of health care reform, China has been working hard to improve the primary health care system, and the government has included diabetes in the national basic public health service projects, including self-management education (27). However, between 2011 and 2015, the rates of patients with diabetes who received health education showed a decreasing trend (28). Generally, the quality characteristics of primary health care in China were poor. Most primary health care was only provided by doctors and nurses in hospitals, whereas the number of primary health care workers was insufficient, and their professional levels were uneven. Therefore, they might be incompetent for diabetes self-management education, which probably explains why patients would bypass them when seeking medical help (29).

How to effectively promote self-management behavior of patients with diabetes is warranted to be explored. American Diabetes Association recommended that the members in the multidisciplinary teams for promoting diabetes education should be involved at least one registered nurse, one registered dietitian nutritionist, or one pharmacist with training and experience on diabetes self-management education, or another health care professional holding as a diabetes Educator or Board Certification in Advanced Diabetes Management (30). They were responsible for patient self-management plans and education such as blood glucose monitoring, exercise, diet, and medication. These plans and decisions were also shared with the primary care provider and reinforced during subsequent visits. Team members negotiated with patients to determine management priorities and action care plans tailored to reflect patients’ knowledge and self-care behavior of diabetes and shared decisions within the team, which improved patient outcomes and reduced the utilization of hospital services (31). Moreover, the composition of team members is flexible. For instance, exercise instructors and psychologists can be involved in physical activity guidance and mental health maintenance if accessible. Non-didactic, interactive and collaborative educational approaches can be adopted to patient-centered or family-based diabetes education. The patient was considered the partner in the discussion rather than the audience of the lecture, which could motivate patients to participate in self-assessment and diabetes education plan development, and transform from passive healthcare recipients to active, empowered and informed health co-producers (32–34).

In addition, digital technologies have been increasingly adopted in studies that focus on improving self-management (35). The utilization of technology provided more resources for motivation, self-monitoring, personalized education, and promoting the homogenization of medical services, which were facilitators for improving self-care behavior and glycemic control, especially in rural areas (36, 37). Meanwhile, there is a need to provide less complex technologies for those elderly patients with impaired cognition and limited technical ability (38). Our study showed that support from spouses and peers was vital for helping elderly patients to maintain self-care. It is also necessary for health care providers to enhance health education on diabetes for family members and peers due to that their knowledge and attitudes toward diabetes are positively associated with patients’ self-care (39–41).

Empty-nest diabetes patients tended to have low utilization of health care services, which was consistent with Zhou (4). With the rapid development of urbanization and the regional imbalance of economic development, the majority of adult children choose to work in provincial capital cities or first-tier cities, usually far from their homes. For the consideration of not bothering their children, who were busy with work, taking care of their families, and far away from home, the elderly didn’t choose to inform their children that they have to go to the hospital until their condition has developed beyond control; this may lead to delays in their disease treatment and have negative effects on their health.

Additionally, financial difficulty was one of the factors affecting the self-care and physical health of empty nesters. The income of the elderly is lower than the average level for the whole province. Many of them have limited pensions and cannot afford medical expenses, especially in rural areas (21). Non-empty nesters living with their children could rely on their children, while empty-nesters did not receive regular and meaningful financial support from children and relatives. Financial difficulty is the leading cause for non-visiting and non-hospitalization, and it exerts a larger negative effect on access to healthcare for empty-nest elderly than non-empty-nest ones (4).

Self-care ability is also a major factor affecting their health (17). In China, care for the elderly is mainly provided by families at home. With the gradual reduction of family size and the disintegration of the traditional large family structure, the function of family endowment is gradually weakened, and the elderly cannot get direct living care (42). Comorbidities are common in older adults with diabetes, more than 97% of them had at least one of the comorbidities (43). Comorbidities not only increase the health care needs, utilization and mortality of people with diabetes (44–46), they also increase the complexity of disease management and the burden of self-management (47, 48) which is a challenge for empty-nester patients to cope with. Decelerated physical function and care for a sick spouse in an empty-nester may be associated with reduced self-care, leading to decreased self-management of diabetes. There is a need for policymakers to shift the responsibility of care from family to public source and establish social security system for the empty-nesters who lack self-care ability and care resources, especially in rural areas.

Older people with diabetes are at a higher risk for psychological challenges such as depression, anxiety, and diabetes distress, and about 14-28% have depression that is two to four times more than the general elderly (49). The incidence of depression in Chinese empty-nesters was 38.6% which was higher than that in non-empty-nesters (50). The departure of adult children, as the most important source of emotional and social support for the elderly, may increase the negative emotions of empty-nesters. Previous studies have shown that compared with non-empty-nesters, empty-nesters are vulnerable to suffering loneliness, anxiety, and depression (6, 51, 52). In contrast, Chang indicated that there was no difference in the incidence of depression between empty-nesters and non-empty-nesters (53). Guo found that the mental health status of the empty-nest elderly was better than that of the non-empty-nest elderly (54). In a longitudinal study of Kristensen, the transition to an empty nest of the family life cycle was shown not to be associated with parents’ psychosocial health in terms of loneliness and depressive symptoms (55). These outcomes may vary with the health status and cultural background of the participants. The inconsistency in research results on the psychological impact of empty-nesting on the elderly needs to be further explored. It is worth noting that social support is an important factor of empty-nest elderly mental health maintenance and diabetes control, especially support from adult children, which seems more important under the influence of the traditional Chinese conception of raising children for the old. However, it is certain that anxiety or depressive symptoms have a negative impact on disease management which can reduce self-care behavior (15). Healthcare professionals and caregivers should pay great attention to the mental health of empty-nest elderly diabetic patients.

Our study findings have some practical implications for the management of empty-nest elderly living with T2DM. Firstly, more diabetes management programs are needed in primary health care, especially in rural areas with poor access to health resources. Secondly, healthcare providers can explore the feasibility and effectiveness of telemedicine in empty-nest elderly living with diabetes, to improve the accessibility to health resources for those who do not seek treatment in time. Thirdly, the role of family support and peer support in disease management can be demonstrated through diabetes education for spouses, peers and adult children, to improve patients’ self-management behaviors. Fourth, strengthen the screening and treatment of depressive symptoms in elderly patients with empty nests. Fifth, it is urgent for policymakers to establish a social security system that can not only provide economic support and medical assistance, but also pay attention to the spiritual needs and social activities of the empty-nest elderly patients to promote health due to the rapid growth of the number of empty-nest elderly and their requirements for services.

## Limitations

There are some limitations to this study. First, this study was conducted entirely in a third-level, grade-A hospital and participants may have a lower self-care than those in a different community. Second, participants were collected from the hospital, and their disease control may be worse than empty-nest elders with diabetes in outpatient and other communities and is limited to other empty-nesters in respect to self-care. In addition, the current study requires a larger sample size and further studies from different clinical/community/social groups are also needed.

## Conclusions

This qualitative study explores the experiences of self-care among empty-nest elderly patients living with T2DM, in terms of blood glucose monitoring, medication compliance, lifestyle modification, prevention of complications, and coping with negative emotions. Empty-nester patients with T2DM perform better in the areas of medication compliance and lifestyle modification, compared with blood glucose monitoring, prevention of complications, and coping with negative emotions. Their self-care behavior is affected by the status of living alone or living with a spouse, which is accompanied by less social support and limited sources of health information. In addition, friends and spouses play an indispensable role in patients’ self-care. Education, access to medical resources, economic and emotional support play an important role in improving their self-management and maintaining mental health under the assistance of health care providers, families, and peers. The findings suggest an urgent need for more diabetes management programs in rural areas for empty-nest elderly with T2DM to improve self-management, as well as for policymakers and health care providers to develop strategies to improve the health status and provide health services for empty-nest elderly with T2DM in the context of aging and urbanization.

## Data Availability Statement

The original contributions presented in the study are included in the article/**Supplementary Material**. Further inquiries can be directed to the corresponding author.

## Ethics Statement

The studies involving human participants were reviewed and approved by Human Research Ethics Committee of School of Nursing, Shandong University. The patients/participants provided their written informed consent to participate in this study.

## Author Contributions

XL made contributions to the conception, design, data collation and interpretation as well as manuscript drafting. DY, YC, and JX were involved in the design, analysis of the data and revision of the manuscript. All authors contributed to the article and approved the submitted version.

## Funding

The study was supported by grants from Department of Science & Technology of Shandong province (2017GSF218001).

## Conflict of Interest

The authors declare that the research was conducted in the absence of any commercial or financial relationships that could be construed as a potential conflict of interest.

## Publisher’s Note

All claims expressed in this article are solely those of the authors and do not necessarily represent those of their affiliated organizations, or those of the publisher, the editors and the reviewers. Any product that may be evaluated in this article, or claim that may be made by its manufacturer, is not guaranteed or endorsed by the publisher.
