# Supplementary material for: Self-Care Experiences of Empty-Nest Elderly Living With Type 2 Diabetes Mellitus: A Qualitative Study From China
Source: Front Endocrinol (Lausanne). 2021 Nov 18;12:745145. doi: 10.3389/fendo.2021.745145 (PMC8636925; doi:10.3389/fendo.2021.745145)
Supplement: Supplementary file 1 [file Table_1.docx]

**Supplementary file**

Self-care Experiences of Empty-nest Elderly Living with Type 2 Diabetes Mellitus: A Qualitative Study from China

Xiaoyan Lv^1,2^, Doris SF Yu^3^, Yingjuan Cao^1,2*^, Jinghua Xia^4^

^1^ Department of Nursing, Qilu Hospital, Cheeloo College of Medicine, Shandong University, Jinan, Shandong province, China

^2^ Nursing Theory & Practice Innovation Research Center, Shandong University, Jinan, Shandong province, China

^3^ School of Nursing, LKS Faculty of Medicine, the University of Hong Kong, Hong Kong SAR, China

^4^ Department of Nursing, Beijing Jishuitan Hospital, Beijing, China

*** Correspondence:**

Yingjuan Cao

Address:Nursing Department of Qilu Hospital of Shandong University, Jinan, China

107 west Wenhua road, Jinan city, Shandong province, China

Phone:+8618560082189

E-mail:caoyj@sdu.edu.cn

**Responses of participants associated with themes and subthemes**

| **Theme and subthemes** | **Illustrative quotes** |
| --- | --- |
| **Self-care deficit in blood glucose monitoring** | |
| Inadequacy in blood glucose monitoring | |
|  | *The primary health care providers test blood glucose for adults with diabetes free of charge every three months, and this is a good choice for me because I feel good about my disease control. (P1)* |
|  | *I test blood glucose about three or four times a year, given that I have been familiar with my glucose control. (P2)* |
|  | *I measure fasting blood glucose once a week in most cases. (P3)* |
|  | *I will record blood sugar for a few days if health care providers ask me to do this. Recording is decreased after this period. (P4)* |
|  | *I measure blood glucose 4-5 times a week, including fasting blood glucose and before sleeping. (P5)* |
|  | *I test my blood glucose every 2-3 weeks. I think my blood glucose meter is inaccurate. (P6)* |
|  | *I tested blood* *glucose when I felt uncomfortable, such as fatigue, dry mouth, and other signs of losing control. (P7)* |
|  | *I test my blood sugar 3-4 times a week, mainly focus on fasting blood glucose. (P8)* |
|  | *I seldom test my blood glucose at home, and I usually go to the clinic to test it about once a month. (P9)* |
|  | *It is unnecessary to test it frequently, I constantly get tested when not feeling well. (P10)* |
|  | *Sometimes I go to the chemist shop near my home to measure it. It's free. (P11)* |
|  | *I measure blood glucose about once a month when it occurs to me. (P12)* |
|  | *I test blood glucose every two weeks in the chemist shop. (P13)* |
|  | *I test my blood glucose every two or three months or every six months. (P14)* |
|  | *I tested my blood sugar every day when I first suffered from diabetes. It's different now. I will not examine my glucose until I feel uncomfortable. (P15)* |
| Barriers for regular blood glucose monitoring | |
|  | *I was tangled about whether to measure blood sugar. I would be happy if it was in my control and depressed when it was high. (P1)* |
|  | *It was common for people with diabetes to feel stressed when faced with high blood glucose, and I asked myself what was wrong with me and what I should do. (P2)* |
|  | *It is stressful to test it, and I'm afraid it is too high to cope with. (P3)* |
|  | *Sometimes I do not want to test my blood sugar; I’m in a terrible temper when the reading rises. (P4)* |
|  | *The test strips were used up so fast, 50 pieces were gone in a few days. It is pretty expensive to buy strips, and needles can be used repeatedly for another time, after first disinfecting with alcohol. (P5)* |
|  | *The workers in the chemist shop test blood glucose twice a month for free, which saves me money. (P6)* |
|  | *I don't think it's necessary to test frequently without discomfort. Otherwise, it will lead to more expenses. (P7)* |
|  | *I resisted the blood glucose test and felt that I had to measure blood glucose again. My fingers swelled because of the blood glucose test. (P8)* |
|  | *I know my condition of blood sugar control, and I don't think it's necessary for me to measure it regularly. (P9)* |
|  | *It is difficult for me to test blood glucose because of visual impairment. (P10)* |
|  | *I think it's harrowing, and I can't do it on my own, making it difficult for me to do it regularly. (P11)* |
|  | *My blood sugar is stable and the test frequency is enough for me. (P12)* |
|  | *I suffer complications with my eyes. It is difficult for me to install the blood taking needle. There is nobody available to help me do this. (P13)* |
|  | *It's very painful for me to measure my blood sugar, so I never test myself. (P14)* |
|  | *It's troublesome to measure blood sugar, and I've been ill for a long time, I don't think it's necessary. (P15)* |
| Inadequate self-care responses to abnormal blood glucose | |
|  | *Perhaps because I am old, going to the hospital to see a doctor is really a big challenge for me, now the hospital and the city is developing so fast and use many intelligent equipment. I will not come to the hospital until my condition is particularly serious. (P1)* |
|  | *I am interested in traditional Chinese medicine, so I searched on the Internet which acupoints can help control* *blood glucose. (P2)* |
|  | *It was hard to keep my blood glucose in a specific range, and I gave up on myself. Whatever, it can't be cured anyway. (P3)* |
|  | *I increased the dose of insulin on my own when the blood sugar was unexpected. (P4)* |
|  | *I adjusted my diet, increased the frequency of blood glucose tests, or increased insulin dose to manage blood glucose levels. (P5)* |
|  | *I ate vegetable noodles when blood sugar was out of control. Once I ate it for half a month, it made me greedy. (P6)* |
|  | *I was used to the fluctuation in blood glucose, and I tried to manage it by eating more vegetables and increasing daily walking time. I will go to the hospital if these do not work. (P7)* |
|  | *I reflected on what I eat or drink, which led to high blood glucose to help me adjusted diet next time. Gradually, I had experience and paid attention to these factors in my diet. (P8)* |
|  | *When hypoglycemia occurred, I ate chocolate. I took it with me when I went out. When hyperglycemia occurred, I ate more vegetables. When the postprandial blood glucose exceeds 15mmol/L, I will see a doctor. (P9)* |
|  | *I am used to hyperglycemia and accept the increase of blood glucose within a specific range. (P10)* |
|  | *Although I learned diabetes knowledge, it was not comprehensive and specific, and some were even incorrect. I didn't know what works when blood glucose was not well controlled. (P11)* |
|  | *I do not know what else to do when the glucose reading exceeds 16mmol. That's terrible. (P12)* |
|  | *I thought I knew little about diabetes, which led to my poor blood glucose management. (P13)* |
|  | *Hyperglycemia and hypoglycemia were both harmful. It was too difficult to control blood glucose within the ideal range. (P14)* |
|  | *I ate the right food, went out for a walk and correctly did everything, but why my blood glucose raised so much. I felt like I couldn't control it. It was frustrating. (P15)* |
| **Self-care deficit about medication compliance** | |
| Adherence to medication order | |
|  | *I take medicine according to the doctor's advice. Sometimes I adjust the dosage myself, such as I overate at dinner with my friends. (P1)* |
|  | *I take medicine as much as the doctor told me to. After all, in addition to controlling blood glucose, it also has side effects. Some friends had hypoglycemia due to increased dosage by themself. It is better to have hyperglycemia than hypoglycemia because you may die from lower blood glucose. (P2)* |
|  | *I take medicine on time, and I will take one more pill to in case of high blood glucose when I eat more (P3)* |
|  | *I often forget to take my medicine and insulin injection, although I have been unwell for a long time. Sometimes I fail to bring an insulin pen with me when I go out. (P4)* |
|  | *When I encounter confusing questions, I will ask my spouse and friends first, because it is more convenient to communicate with them, and the doctor is far away from me. (P5)* |
|  | *I take xiaoke pills to control my diabetes, five in the morning, five in the evening and metformin at noon. (P6)* |
|  | *I ate acarbose with water. Later I knew it should be chewed. None of the original drugs worked due to improper use, and my health care providers never told me before. (P7)* |
|  | *I have to take several kinds of medications that sometimes I forget whether I take them or not. (P8)* |
|  | *I take medicine discontinuous and adjust the dose according to my blood glucose level. (P9)* |
|  | *My husband needs rehabilitation exercise because of stroke. I need to spend much time helping him, which makes me often forget to inject before eating. (P10)* |
|  | *I rarely forget to take my medicine. (P11)* |
|  | *My spouse is hemiplegic. Sometimes when I am busy taking care of him, I forget to take my medicine. I am the only one in the family who can't cope with it at some time, not to mention also managing diabetes. (P12)* |
|  | *My blood glucose was well controlled when I stopped taking medicine, so I didn’t eat anymore. (P13)* |
|  | *You can ignore any other management strategy other than taking medication. Taking medicine is the most essential way to control blood glucose. (P14)* |
|  | *My stomach was uncomfortable, and I had diarrhoea after taking metformin, then I dropped out. (P15)* |
| Using health products to supplement the action of medications | |
|  | *I used to put my faith in health products, and now I don't believe in them at all. A person in our community was diagnosed with [kidney](javascript:;) [stone](javascript:;) because of eating healthy products. My son and daughter reminded me not to eat them in case of health injury and to take medicine on time. (P1)* |
|  | *As I get older, I feel useless. I can't help with my children's affairs, and I don't want to bother my children with trifles. What I hope for the most is that there are some ways to cure diabetes, so that I can suffer less and be less trouble for my family. (P2)* |
|  | *I don't believe in health products. It's expensive and useless. I'd better listen to the doctor. (P3)* |
|  | *If taking medicine can't control blood sugar well, I don't think taking health products has any effect. (P4)* |
|  | *I've heard that some health products claim to lower blood sugar, but the actual effect is unknown. A friend bought it; however, blood sugar became higher after taking it. It is not easy to guarantee the effect. (P5)* |
|  | *I expect to cure diabetes. My husband heard that a drug with sound effects, and I thought I would try it. However, it did not work for me, and my daughter emphasized that it was a lie. (P6)* |
|  | *Health products do not have any effect and also have no harm to our bodies. It makes no difference whether you eat them or not. Maybe some people take it for psychological comfort. (P7)* |
|  | *She (a neighbor) told me that her blood glucose level dropped after taking health products without any diabetes medication intake. I spent 5 thousand RMB to buy it for its good effect. I took it for 2 months, and there was not any improvement in my blood sugar. I haven’t finished it yet. (P8)* |
|  | *I don't like taking medicine, let alone health products.* *Every medication has its side effect. If the doctor didn't recommend it, I wouldn't try. (P9)* |
|  | *Health products are all over the place where I live. The products are fake and sellers persuade you to buy these types of things all the time. He (spouse) heard of drug ads for Tongrentang on the Internet. You know, the brand is famous (in Beijing). Tongrentang is a famous time-honored brand in the traditional Chinese medicine industry, and we trust it. I spent about 1380RMB buying six bottles of medication, attempting to control my blood glucose level. It turned out to be ineffective in the end. (P10)* |
|  | *Regardless of the effect, it is pretty expensive, dozens of times more than the drugs I use. I can't afford it. (P11)* |
|  | *I used to spend money to buy fish maw and sheep placenta to cure diabetes at the recommendation of friends, but it was useless. My daughter said I should get her opinion before bought health products. (P12)* |
|  | *Diabetes could have been cured a long time ago if health products worked as advertised. (P13)* |
|  | *I thought about buying health care products before because some people said the effect was excellent. My children told me that it was all a gimmick, and I never talked about it again. (P14)* |
|  | *Insulin injection is enough for me. It is a high risk for me to have hypoglycemia if I also eat health products. (P15)* |
| **Lifestyle modification** |  |
| Being empty-nesters - a facilitator for diet management | |
|  | *I took their preferences as a priority and ate the same with them, and I had no more energy to prepare another different meal simultaneously. (P1)* |
|  | *When I lived with my son, he cared about my blood sugar control, reminded me to eat less animal offal and cook with fewer condiments, which pushed me to do better. (P2)* |
|  | *I tried to control my diet as much as possible, but you know, I inevitably face too many temptations to eat with them and break the restrictions. (P3)* |
|  | *I eat the same as the children do when they are back home, and I eat more coarse grains for breakfast and dinner. (P4)* |
|  | *I had to do housework and picked up my granddaughter. I didn't have time to consider my dietary restrictions when I lived with them. (P5)* |
|  | *Living with children or not has little impact on my diet, I always eat what I want. (P6)* |
|  | *Well, I tried to control my mouth, but there were too many temptations, and sometimes when my daughter-in-law cooked, I couldn't ask her to prepare suitable food for me. (P7)* |
|  | *I told my daughter not to buy me any food, I could buy whatever I wanted. I didn't think they understood my dietary restrictions. (P8)* |
|  | *It is easy to manage my diet without living with children. Some of my children like eating meat; also my grandchildren need to grow up healthy and require foods that I am restricted from. (P9)* |
|  | *I don't have to worry about them. I have my own time to do what I want to eat and what I should eat. (P10)* |
|  | *My children bought food that exceeded my dietary restriction, and they thought it couldn't affect diabetes management. It was hard to refuse. I didn't have to worry about that when I lived without them. (P11)* |
|  | *My son was busy with work. I took the responsibility of taking care of his family when I lived with him. I did whatever they like to eat. I didn't have such high requirements for myself. I tried to eat less rice and fruit to control my blood sugar. (P12)* |
|  | *My blood glucose is what I eat, so I pay more attention to diet restrictions whether I live with my son or not. (P13)* |
|  | *I don't have restrictions on diet. I like porridge and food with a heavy taste. Food is the first necessity of the people. It's challenging to insist on eating light every day. (P14)* |
|  | *I don't have much control over eating out with other people. If you adhere to dietary restrictions when eating out, it is considered that you are picky with food and out of tune with others. (P15)* |
| Compliance with physical activity | |
|  | *I walk for half an hour in the morning and half an hour in the afternoon. (P1)* |
|  | *I do Tai Chi for half an hour with my friends most of the morning. I also suffered from myocardial infarction and can't do an intensive activity. (P2)* |
|  | *I don’t exercise regularly. I usually do housework at home. I go out for a walk when there is nothing to do at home. I used to do exercise and now, it is difficult for me to walk too much since I broke my ankle five years ago. (P3)* |
|  | *I go out walking or biking for an hour almost every day. (P4)* |
|  | *My husband accompanies me with my exercise, even in winter. I can’t insist on doing that without him. (P5)* |
|  | *I dance with my neighbours for an hour every morning, and she also has diabetes. I also walk for 1.5 hours after dinner. (P6)* |
|  | *I walk in the community for 40 minutes almost every night after dinner. (P7)* |
|  | *I got up at six o 'clock in the morning and went out for a walk with my husband. I finished at about seven o 'clock and went home for breakfast. (P8)* |
|  | *I have a meniscus injury and activity limit, but I still go for a walk half an hour after breakfast, which is about 2 kilometres. (P9)* |
|  | *I walk 5 or 6 laps, at least, around the square every afternoon. I go out at 2 p.m. and am back home at about 5 p.m. I insist on walking 2.5 hours a day, approximately, unless there is severe weather. (P10)* |
|  | *When the weather is warm, I like to go out for half an hour. I don't go out when the weather is cold, such as in winter. (P11)* |
|  | *I need to do housework and have no time to go out for exercise. (P12)* |
|  | *I have arthritis, which limits my activities. I can't walk and run fast. (P13)* |
|  | *I like swimming, and I make an appointment with my friends twice a week for about an hour each time, which makes me feel energetic. (P14)* |
|  | *I often go out for a walk with my friends, or with my spouse. They give me a lot of companionship and encouragement. I think it's hard for me to keep going out for exercise almost every day without their company and supervision; they are important to helping me have the motivation to exercise. (P15)* |
| **Self-care deficit in diabetes** | |
| Limited available information sources | |
|  | *I haven't heard of any community lectures, and I wouldn't attend them even if I had. I attended a lecture before, which was given by an old nurse. I couldn't understand what she was talking about. She left after the lecture without any interaction. Some of them are just there to make money. The quality of lectures is poor, and I don't want to listen again. (P1)* |
|  | *I watch Honyaradoh every day, which makes me have a deep understanding of diabetes and some Chinese medicine health care methods. (P2)* |
|  | *My mother has diabetes. I learned about diabetes from her; moreover, I receive knowledge from HCPs when I go to the hospital or community clinic. (P3)* |
|  | *I watch TV and listen to lectures at home. “The Road to Health” is the only China Central Television talk service program focusing on the public's physical and mental health awareness and advocating a healthy life. I’d like to watch this at home. (P4)* |
|  | *Doctors in the community didn’t explain so much about managing diabetes. Sometimes I wonder if I am doing the right thing to improve blood glucose levels. I hope to get the guidance of a professional doctor. (P5)* |
|  | *When I was in hospital, I listened to lectures and watched health programs at home for knowledge. Nevertheless, I still felt that I didn't know much about diabetes. I want to learn more about how to control the process of diabetes. In addition, I find it challenging to translate knowledge into practical action. (P6)* |
|  | *Primary health care provides free blood sugar testing for diabetics. The doctor told me to adjust diet and increase exercise when blood glucose was out of control, but I was still confused about doing it in detail. (P7)* |
|  | *I was used to watching lectures on diabetes on TV, and I used to work in the health system, so I know how to manage diabetes. The primary care physician in my community also called to ask about blood sugar control, and I consulted him when I had problems. (P8)* |
|  | *I had never received professional diabetes education until I was in the hospital. Face-to-face communications helped me obtain more management skills. (P9)* |
|  | *Our community held diabetes lectures, but I had not been there. I learned knowledge about diabetes through watching TV or searching by mobile phone. (P10)* |
|  | *I don't think I have a reliable source of information. Before I was hospitalized in the county hospital, the doctor taught me to control blood sugar, but there was no effect. I found that their opinions conflict with those given by the doctors here, I prefer to believe in the doctors here. (P11)* |
|  | *I watch TV, listen to lectures, watch my mobile phone, and learn about diabetes, but I can't identify which ones are effective. (P12)* |
|  | *When I was in hospital, doctors and nurses introduced diabetes management and taught dietary adjuestment and exercise. When I got home, I didn't know how to do it. There was no guidance, and I didn't know whether it was right or not. (P13)* |
|  | *I searched the Internet for the effect of fruits on blood sugar and then decided whether to buy it or not. No attention had been paid to other diabetes information. (P14)* |
|  | *I am close to the hospital, I go to the hospital when my blood sugar is high. Diabetes knowledge is learned when I go to the clinic or in hospital. (P15)* |
| Poor knowledge about complications | |
|  | *I never knew that diabetes would cause so many complications until I was in the hospital. (P1)* |
|  | *I think I know enough about diabetes. It can damage other organs, such as eyes and feet, so controlling blood sugar is still essential. (P2)* |
|  | *I don't know what examinations should be done to prevent or screen early changes, and I never do screenings about related complications. (P3)* |
|  | *I wash my feet every night, massage them to promote blood circulation and observe for any lesions. (P4)* |
|  | *I don't have much confidence in diabetes management. I suffered from weak legs and diabetic retinopathy. It is too late for me to understand the complications of diabetes. (P5)* |
|  | *An elderly relative of mine also had diabetes and diabetic foot ulcers, and it looked too scary. I don't know what diabetes will bring; all I learned was to keep blood glucose under control. (P6)* |
|  | *I didn't know the complications of diabetes before, nor did I know how to deal with them. I have no discomfort. (P7)* |
|  | *I understand the harm of diabetes, and I try my best to adjust my lifestyle and learn more about diabetes management skills. My daughter often sends me some articles related to diabetes. (P8)* |
|  | *I used to know little about the complications of diabetes, but I've got eye complications now, and it's hard to see things (frown). (P9)* |
|  | *Diabetes can cause rotten feet, I am terrified that it occurs to me. I can’t understand why feet can be damaged by diabetes. (P10)* |
|  | *Because of this hospitalization, I was diagnosed with peripheral neuropathy, and the sensation of my feet was decreased. Just now, the nurse introduced to me how to protect my skin and how to check my feet. I felt I knew nothing about diabetes at that moment. (P11)* |
|  | *Proteinuria was found during a physical examination, and I was diagnosed with diabetic nephropathy in hospital. I was surprised that diabetes was devastating and at a loss as to what happened. (P12)* |
|  | *I learned that the greatest harm of diabetes is complications. Blood glucose control is the most important thing. (P13)* |
|  | *I think that diabetes should not be regarded as a disease. It doesn't restrict my diet nor delay my work. In other words, I live my life as usual. (P14)* |
|  | *Through hospitalization, I realized that it was mainly to control myself from diet and strengthen exercise. I can't be lazy. I have to keep better diabetes management in order to prevent diabetes from deteriorating. (P15)* |
| **Coping with negative emotions** | |
| Negative emotions associated with diabetes | |
|  | *It's hard to avoid being in a lousy mood. I adjust myself and learn to treat diabetes with positive emotions. After all, sighing all the time doesn't change anything. (P1)* |
|  | *I can't relax after being diagnosed with diabetes. It is always in my mind. (P2)* |
|  | *I have to change my lifestyle for better management. That's too much trouble. (P3)* |
|  | *Patients with diabetes suffer from great stress when their blood glucose is out of control. (P4)* |
|  | *When I went out to dinner with friends, I considered which food was beneficial to glycemic control. I injected insulin in the corner because I didn't want to be treated differently from others. (P5)* |
|  | *Diabetes does bring much inconvenience to my life, but what I can do is accept it and manage it according to the doctor's advice to benefit from it. Otherwise, the pain will still be my own. (P6)* |
|  | *More attention should be paid to the mental health of people who were newly diagnosed. They faced more worry and anxiety. (P7)* |
|  | *The last time I sick, I was thinking about how hard it is for humans from birth to death. I suffer living with diabetes. (P8)* |
|  | *I am an old person with diabetes, which makes me feel useless. (P9)* |
|  | *I get upset and don't want to communicate with others when my blood sugar is not under control (P10)* |
|  | *I felt upset and annoyed after I recovered from hypoglycemia. It was hard to know what would happen. (P11)* |
|  | *I have to do housework and take care of my husband. I'm too tired to take care of diabetes management. (P12)* |
|  | *Sadness happens from time to time, I try to recover quickly from it and face it. (P13)* |
|  | *When I was diagnosed with diabetes, I thought about curing it every day and went to different hospitals for treatment. I had no appetite when high blood glucose occurred on me. Now I believe life is short and do what makes you happy. (P14)* |
|  | *It is suffering to have diabetes, you must pay attention to your diet, physical activity, and insulin injection. It is imbalanced compared with the responsibilities other people have (sigh). (P15)* |
| Psychosocial support from family, peers, and healthcare professionals | |
|  | *My husband gives me much support. He reminds me to take medicine and does exercise with me. He is more concerned about my glycemic control than me. (P1)* |
|  | *He (spouse) spends most of his time playing cards, watching TV, and doing other things that he is interested in, rather than paying attention to my disease. (P2)* |
|  | *When I'm feeling down, I talk to my friends. They encourage me to do better. They also patiently listen to me. It makes me feel like I’m not alone in the face of diabetes. (P3)* |
|  | *Because of diabetes, I visit the psychological clinic. Dr Li listened to me patiently and understood the negative emotions brought by diabetes. Meanwhile, she tried to enlighten and encourage me. I firmly believed that this was necessary to do that, no one would be willing to listen to my negative emotions except them. (P4)* |
|  | *Negative things let everyone felt annoyed, and I bear most of them by myself. (P5)* |
|  | *I have several friends with diabetes. We shared difficulties, skills, and experiences. I was relieved after talking with them. (P6)* |
|  | *I have high self-esteem, and I do not want to show my weakness to others. Moreover, I do not like to tell anyone about my condition. (P7)* |
|  | *I adjust myself sometimes or express my feelings to my daughter. She blames me all the time. She is busy and stressed with her life, and I don’t want to trouble her too much…Our children treat us well and always buy us something to eat. Those things are unsuitable for me. The most common thing they say to me is to take my medicine on time and to exercise more. They always say that. (P8)* |
|  | *I want more attention from my kids, my friends, my doctors. But they don't seem to have that much time to listen to my messy little things, and sometimes I don't think they can put themselves in my shoes. (P9)* |
|  | *My children blamed me for overthinking and thinking about something out of order all day long when I complained to them about diabetes. (P10)* |
|  | *I went to the psychology department of a traditional Chinese medicine hospital when I felt depressed. I felt so much better after talking with psychologists. I prefer talking with them. Sometimes you need somebody who listens to you like this. (P11)* |
|  | *My son gives me a call at least once a week to ask me about my blood glucose control and medication, and ask me to control my diet and give me 2000 RMB a month. Although he is not with me, I think he have done well. (P12)* |
|  | *Who can truly understand what I feel except for myself? If they ask me how I'm doing, I tell them not to worry, I'm fine. (P13)* |
|  | *My wife thought that diabetes was not a severe illness. She never cared about it. I didn't want to complain to her about it. It may make us feel annoyed. (P14)* |
|  | *I don't like to share my troubles with others. I told my children that people with diabetes suffered too much. They said it was common to have the disease, and they were annoyed when I said more. I also try to keep a peaceful mind, and can’t be frightened by the condition. (P15)* |
